# Disappearance of Dr. Parkman, of Boston, Mass. and Arrest of J. W. Webster, of the Mass. Med. College, on the Charge of Murder

**Published:** 1850-01

**Authors:** 


*Disappearance of Dr. Parkman, of Boston, Mass., and arrest of Prof. J. W Webster, of the Mass. Med. College, on the Charge of murder.*—The above events, which have astonished and shocked the country and the world, claim to be noticed by the Medical Journalist from the connection of the individuals named with the Medical Profession, the latter occupying a distinguished position as teacher of a collateral branch of medical science in one of the oldest, and most respectable of the medical institutions of the Union. With all the circumstances, in so far as these are known to the public, our readers are doubtless acquainted. We have, of course, nothing to add to what has been communicated by means of the newspaper press. The identification of the remains found in, and beneath the laboratory of Prof. Webster, is stated to be complete, although the evidences of this conclusion do not fully appear in what has been reported. Prof. Webster is now confined in jail, on the verdict of a coroner's inquest charging upon him the crime of murder. The case will come before the grand jury during the present month, and should an indictment be found, it is stated that the trial will probably take place in May next. What the result will be, it is, of course, gratuitous to predict; nor would it be becoming in us to express any opinion to the innocence or guilt of the accused. But this we may say, as we conceive, with propriety, and, if we mistake not, the remark is called for;—The unblemished character of a man, over fifty years of age, holding a high intellectual and social position, should shield him from premature conviction in the minds of his professional brethren, and the public. So much as this at least, should be awarded, not only in charity, but in justice. It is to place a small value upon a life unsullied by vice, to refuse the simple boon of a suspension of judgment until a legal tribunal has pronounced a verdict. For one, if an opinion must be entertained, without an investigation of the evidence which a fair trial can alone develop, we would, by far, incur the risk of erring in refusing to believe that such a man could be guilty of such a crime, until his guilt be clearly established. Upon careful examination of all that has as yet been published relative to this (for both parties) tragical event, it will be found that nothing appears incompatible with acquital, not only of the charge, but of all suspicion. Should this be the result, and God grant that it may be so! It is to be hoped that the authors of a conspiracy hardly equalled in the annals of crime, may not escape the guilt of a two-fold murder, and that Prof. Webster may have no cause to reproach his professional brethren for not exercising that Christian virtue which "hopeth all things," "thinketh no evil," and "never faileth."

